# Claim for Rebate of Spirit Duty

**Published:** 1917-03-17

**Authors:** Jas. M. Churchfield

**Affiliations:** Secretary. St. George's Hospital, S.W., March 7, 1917


### Claim for Rebate of Spirit Duty.

*To the Editor of* **The Hospital.**

Sir,—With reference to the note in your issue of the 24th ult., *re* the above, it may interest you to know that, on application being made to the Local Government Board on behalf of this hospital for the necessary form of application, a reply was received from that Department as follows :—

"The form of application for grants in relief of voluntary hospitals in respect of duty paid on spirits used for medical and surgical purposes is in course of preparation, and copies will be forwarded in due course."—Yours faithfully,

Jas. M. Churchfield, Secretary.

St. George's Hospital, S.W., March 7, 1917.

